# Gendered preferences for varieties and traits in Uganda’s common bean value chain: insights for breeding programs

**DOI:** 10.1057/s41599-026-07472-w

**Published:** 2026-05-22

**Authors:** Eileen Bogweh Nchanji, Collins Odhiambo Ageyo, Wilber Ssekandi, Grace Nanyonjo, Grace Bantebya, Cosmas Kweyu Lutomia, Victor Nyamolo

**Affiliations:** 1 International Center for Tropical Agriculture, Nairobi, Kenya; 2 National Agricultural Research Organisation, Kampala, Uganda; 3 Makerere University, Kampala, Uganda; 4 Egerton University, Nakuru, Kenya

**Keywords:** Economics, Anthropology, Science, technology and society

## Abstract

Common bean breeding efforts have focused on understanding and developing product profiles that align with farmers’ trait preferences with limited emphasis on downstream value chain actors. Varieties that do not consider client-preferred traits are often on the shelves and not adopted by the targeted clients. Thus, considering trait preferences of downstream bean value chain actors like processors, traders and consumers is becoming important as they sometimes differ from those of farmers. This study investigated gender varietal and trait preferences of traders, consumers, and processors for common bean varieties. A mixed method was used to collect data from 75 traders, 21 processors, and 100 consumers in Uganda. Descriptive results identified that short cooking time, thick stew, good taste, and affordability are intertwined and the most preferred traits. Irrespective of gender, actors preferred existing varieties such as NABE 4, Kanyebwa, Masindi Yellow, Nambale Omuwanvu, and NARO BEAN 1 for possessing the most preferred traits. These findings can guide common bean breeders in targeting these traits to meet the needs of men and women processors, traders, and consumers.

## Introduction

Common bean (*Phaseolus vulgaris* L.) is the most affordable and widely consumed source of protein in sub-Saharan Africa, providing slow-digesting carbohydrates that are packed with essential micronutrients such as iron and zinc (Njuki and Andersson, 2014; Binagwa et al., 2019). In Uganda, common bean is a strategic food and income security crop, prioritized under Uganda’s Agriculture Sector Strategic Plan (Ministry of Agriculture, Animal Industry and Fisheries, 2019), and cultivated by over 60% of smallholder households predominantly women contribute about 57% of the labour and play a leading role in variety selection and seed management (Esuma et al., 2019). Common bean production in Uganda occupies an average of one million hectares of land per year, ranking second after maize (Larochelle et al., 2017). Beans are consumed in virtually every household, with per capita consumption ranging from 50 to 60 kg per year) particularly among low-income rural and urban consumers (Larochelle et al., 2015).

Although the crop significantly contributes to poverty and hunger reduction, common bean production remains constrained by climatic stressors, declining soil fertility, and diseases such as bean rust and angular leaf spot (Kimiti, 2014). In response, bean breeding programmes, most notably by the Alliance of Bioversity International and the International Centre for Tropical Agriculture (Alliance) through the Pan African Bean Research Alliance (PABRA), have released multiple drought-tolerant, disease-resistant varieties, and short-maturing varieties in recent years (Mukankusi et al., 2015). These breeding efforts aim to enhance climate resilience and productivity. However, concerns persist concerning the over-prioritization of agronomic traits most emphasized by farmers over the preferences of downstream value chain actors. This may unintentionally influence the adoption of developed common bean varieties, affect market performance and consumer acceptance altering the expected impact (Nchanji et al., 2021). Additionally, despite widespread promotion of improved varieties, adoption has remained modest in some areas due to misalignment with market and consumer preferences (Asiimwe et al., 2024; Nchanji et al., 2021).

Trait preferences are increasingly recognized as gendered and context-specific (Assefa et al., 2014). Existing studies reveal that farmers’ varietal and trait preferences are shaped by intersecting agronomic, sociocultural, and non-market factors, such as maturity period, yield, and resistance and tolerance to biotic and abiotic stresses, which have been identified to influence gendered varietal preferences (Assefa et al., 2014; Mulu et al., 2017; Nchanji et al., 2021). Gender roles and intra-household decision-making structures often determine who selects varieties and why (Assefa et al., 2014). However, most studies on varietal and trait preferences have narrowly focused on farmers’ preferences, with limited attention on how other value chain actors, such as consumers, traders, and processors (e.g., Alemayehu Balcha and Rahel Tigabu, 2015; Nchanji et al., 2021), evaluate bean varieties and traits such as grain affordability, taste, and cooking time as identified in a few studies (Asiimwe et al., 2024; Nchanji et al., 2023).

Recent scholarship has emphasized the need to align breeding priorities with the demands of multiple value chain actors (Nakazi). Although it is considerably challenging to incorporate all traits in breeding product profiles, demand-led and gender-responsive breeding have emerged as critical frameworks that advocate for integrating preferences across value chain nodes to ensure varietal uptake and impact (Ciro Dominguez, 2023; Ndabashinze et al., 2024). Demand-led breeding is designed to incorporate trait preferences of diverse actors in the development of product profiles to address the needs of multiple actors in the bean value chain. Gender-responsive breeding underscores that trait preferences are not only actor-specific but also gender-differentiated, intersecting with education, marital status, age, and occupation to shape varietal demand (Ashby and Polar 2021; Orr et al., 2021; Lawali et al., 2024; Ndabashinze et al., 2024). Addressing these complexities requires participatory methods that go beyond farm-level actors.

Participatory varietal selection (PVS) is a breeding and social approach involving farmers in identifying materials and traits that must be incorporated into breeding programmes. Breeders have traditionally used this approach to identify context-appropriate varieties by incorporating farmers’ experiential knowledge and trait preferences in variety development (Temesgen Begna, 2022). However, the approach has not consistently succeeded in engaging downstream actors in the value chain. Therefore, there is growing recognition that such approaches should be expanded to include end-users and market intermediaries (Assefa et al., 2014; Zeleke et al., 2025). While traders and processors are vital in determining the commercial viability of product profiles and consumer acceptance of varieties, few breeding initiatives systematically elicit their preferences. Studies that have integrated traders’ and consumers’ perspectives, such as in cassava and sweet potato breeding (Kikulwe et al., 2021; Thiele et al., 2021), reveal significant gaps between farmer traits and end-user expectations. Thus, breeding programmes need to extend PVS beyond farmers by involving traders, processors, and consumers in selecting varieties. Involving downstream actors in PVS would ensure that their varietal preferences and traits are incorporated into breeding programmes.

Trait and varietal preferences also differ by gender, intersecting with other socioeconomic factors such as education, age, marital status, income level, and household roles to shape differentiated needs and choices (Croson and Uri, 2009). We argue that the intersecting elements may influence men’s and women’s varietal preferences. Although there has been increasing focus on gender-responsive and demand-led breeding, available empirical studies have a narrow focus on farmers’ varietal preferences and how they are shaped by socioeconomic variables (Nchanji et al., 2021). This study extends existing literature by examining how bean varietal preferences among traders, processors, and consumers are shaped by gender in combination with intersecting socioeconomic factors.

The findings highlight differentiated priorities across actor profiles that are useful inputs for bean breeding programmes, particularly in designing varieties that respond to diverse end-user needs shaped by gender, market roles, and intersecting socioeconomic factors. The study examined the hypothesis that varietal and trait preferences among downstream actors vary systematically and may vary by gender and intersecting socioeconomic factors, with the quantitative patterns interpreted as exploratory, given the modest and uneven subgroup sizes.

## Methodology

### Study area

**Fig. 1:**
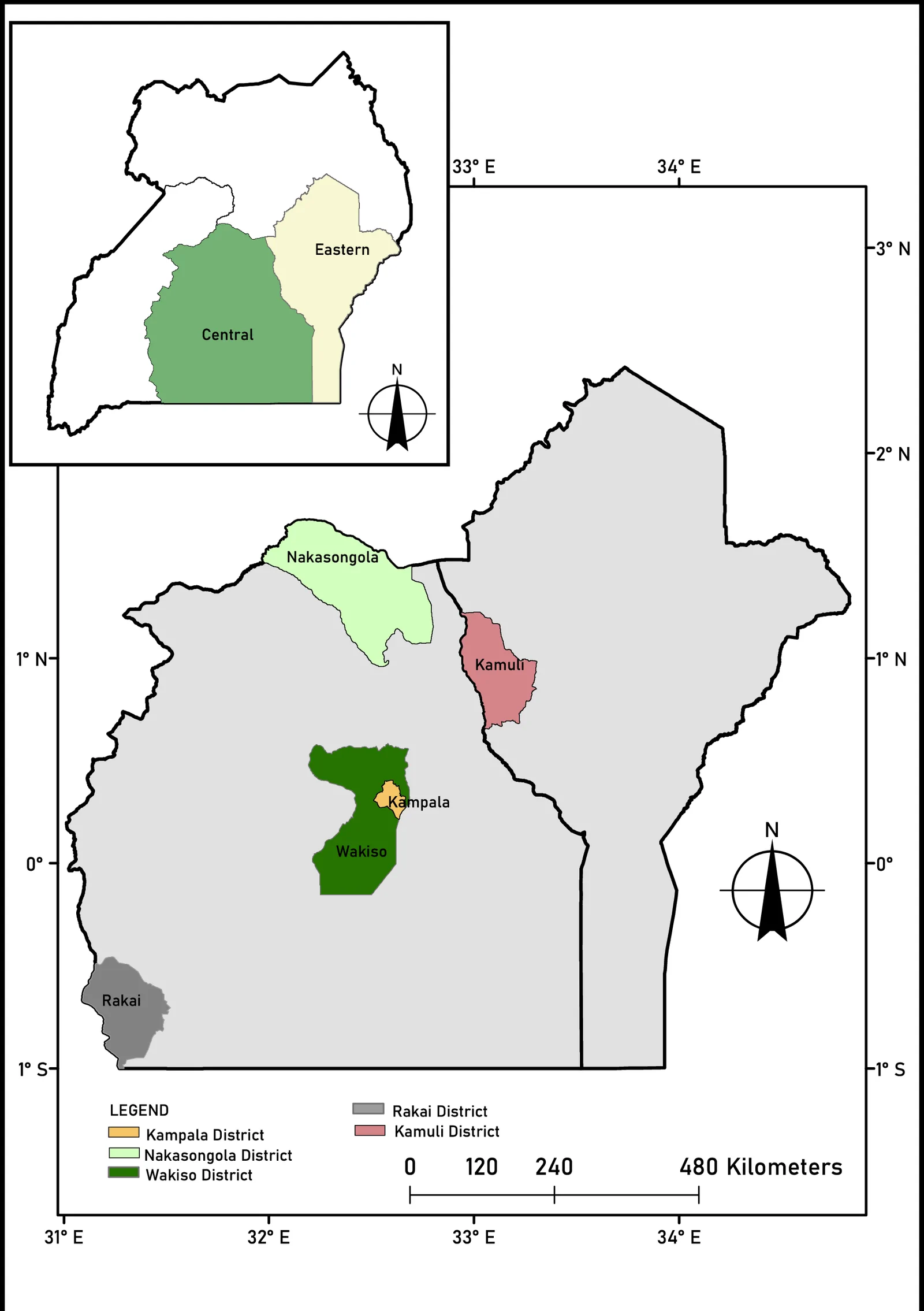
Map of the study area showing the five bean-producing districts involved in the study. Authors’ map.

The study was conducted in 2020 in Nakasongola, Rakai, Wakiso, and Kampala districts in Central Uganda and Kamuli district in Eastern Uganda, as shown in Fig. 1. These districts are characterized by a tropical climate favourable for common bean production. The districts receive bimodal rainfall, with the main season starting in April to October and the short season from November to March (Ministry of Agriculture, Animal Industry and Fisheries (MAAIF, 2019). Farmers in the five districts grow improved common bean varieties, mainly released and disseminated by Uganda’s National Agricultural Research Organization (NARO), Action for Fundamental Change and Development (AFFCAD), and NAADS programmes.

### Sampling design

A two-stage stratified sampling method was used to identify the study samples. The study purposively selected Eastern and Central regions because they are among the leading bean-producing regions in Uganda, and we selected five districts from these regions. The five districts, four in the Central region—Nakasongola, Rakai, Wakiso, and Kampala districts and Kamuli in the Eastern region, are the major intervention districts of the Pan African Bean Research Alliance (PABRA) and the National Agricultural Research Organization (NARO) bean programmes. Stage two involved the selection of villages and farmers’ names from lists obtained from the district extension officers.

Group leaders were requested to provide lists of traders, processors, and consumers, which were entered into Excel to form three distinct sampling frames. Names were randomly selected using Excel, and probability proportional to size sampling was applied to determine the number of participants selected per district. The final sample included 100 consumers (79 women and 21 men),75 traders (40 women and 35 men), and 21 processors (20 men and 1 woman). This disparity in the representation of women among processors is not the result of sampling oversight but rather indicative of broader gendered dynamics in the bean processing segment, shaped by structural inequalities. Among consumers, 67 women and 17 men had previously participated in PVS. In contrast, only one male processor reported prior participation in PVS, and none of the traders had ever attended a PVS exercise.

However, for processors, the sample comprised predominantly men (20:1 male-to-female ratio). This disparity reflects the actual gender distribution in bean processing enterprises in Uganda, where men tend to dominate due to ownership patterns, capital requirements, and machinery operation, which remain structurally inaccessible to many women. This contextual reality is acknowledged as both a finding and a limitation, underscoring the need for targeted support to enhance women’s participation in higher nodes of the value chain.

### Data collection and analysis

The study used a mixed-methods research design. Quantitative data were collected using a semi-structured questionnaire, which collected information on respondents’ socioeconomic characteristics and varietal preferences and traits. The PVS data collection tool was used to collect trait data. The traits included in the structured PVS tool were adapted from a pre-validated set of traits previously used in PVS exercises, sensory evaluation, and breeding programme feedback loops. Captured traits of interest included taste, stew thickness, grain size, marketability, affordability, availability of the common bean grain, and cooking time etc. The traits were tied to each bean variety that was a PVS candidate. The study used descriptive statistics such as means, percentages, and frequencies to explore how varietal and trait preferences compared by respondent type, gender, and intersectional elements, aiming to describe observed patterns rather than to make statistical inferences about trait dimensions. Given the modest and uneven subgroup sizes across value chain nodes, the quantitative results are presented as exploratory descriptive patterns.

Qualitative data were collected through four sex-disaggregated focus group discussions (FGD)that were conducted in Wakiso and Kamuli districts, each involving 6–8 consumers. The discussions were held in local languages, audio-recorded, translated, and transcribed verbatim into English. The transcripts were then subjected to thematic analysis. The coding framework was developed based on the study’s key research questions. The codes were refined and those generated inductively during close reading of the transcripts. Thematic patterns were identified, categorized, and compared across gender groups to capture divergent and convergent perspectives on varietal and trait preferences. The analysis process was conducted manually to ensure that contextual interpretations of narratives were preserved and reported.

## Results and discussions

Table 1 presents the socio-demographic characteristics of sampled respondents by gender. Given the small and uneven subgroup sizes in some value chain nodes (notably processors), the descriptive statistics in Table 1 and subsequent tables should be interpreted cautiously, since single observations can shift the reported shares. Among processors, the sample was overwhelmingly male (20 men, 1 woman), and male processors were relatively young (mean age 24.1 years), while the female respondent was 45 years. The National Development Plan II (2015/2016–2019/2020) made common bean a critical commercial crop in Uganda after banana, maize and coffee. This might have influenced young men to venture into bean processing (Nakazi et al., 2017). Furthermore, bean processing attracts more young men because it provides employment opportunities to the country’s unemployed youth (Mutenyo et al., 2022). The average age of women and men traders was about 35 years. This finding reinforces the view that common beans have the potential to create job opportunities for working-age adults, including youth in Uganda, a perspective supported in the literature by Mauyo et al. (2010) and Tamusange et al. (2020), who recognize the crop as a potential cornerstone for the Ugandan economy.

**Table 1:** Socio-demographic characteristics of the sampled respondents disaggregated by gender.

	Traders (*N* = 75)		Processors (*N* = 21)		Consumers (*N* = 100)	
Variable	Women (*n* = 40)	Men (*n* = 35)	Women (*n* = 1)	Men (*n* = 20)	Women (*n* = 79)	Men (*n* = 21)
Average age of the respondent	34.63	34.83	45.0	24.1	47.65	49.00
	(12.11)	(9.87)		(5.2)	(14.38)	(12.62)
Household head (%)	32.50	74.29	0.00	75.00	43.04	95.24
Marital status (%)						
Married	65.00	57.14	100.00	50.00	64.56	90.48
Never married	22.5	37.14	0.00	45.00		
Widowed	10.00				22.78	
Separated		5.71			10.13	
Divorced					1.27	
Cohabiting	2.50		0.00	5.00	1.27	9.52
Education level (%)						
No formal education					7.59	0.00
Primary	25.00	22.86	100.00	80.00	67.09	61.9
Secondary or higher	75.00	77.14	0.00	20.00	25.32	38.1
Off-farm employment as main occupation (%)	97.30	81.25	1000	95.00	10.13	0.00
Average household size (number)	5.53	4.71	5.00	2.75	7.61	8.24
	(2.44)	(3.18)		(2.17)	(3.76)	(3.05)

The study defined the household head as the person the respondent identified as the household head, which typically reflects socially recognized authority and final decision-making power in the household. Results in Table 1 show that more than 74% of respondents reported living in male-headed households. This result is expected given the strong sociocultural values that position men as de facto household heads. Even where women manage day-to-day food and consumption decisions, prevailing sociocultural norms often lead households to report men as the household heads and final authorities in food-related matters in some contexts. For instance, Michelle Hindin (2005) reported that women in Malawi, Zambia, and Zimbabwe lacked autonomy in making nutrition decisions. On the other hand, men’s domination of household headship among traders and processors could be associated with their relatively greater financial endowment compared to women. In addition, men tend to target high-value chain nodes, while women dominate less remunerative nodes. It is critical to note that consumers were mostly farmers; therefore, women were likely engaged in production and farmgate-level marketing. This assertion was further reiterated in an FGD by a male respondent who stated that:

Madam, no man of this group has participated in those gardens you talked about. That work is for women who have time to waste. Most of us have heard about those demonstration gardens, but we are not interested in participating. They involve many activities, such as weeding, land preparation, spraying, monitoring, and many others. They take a lot of time yet fetch no monetary value. Also, we are responsible for taking care of our families. We must look for a way to survive and engage in activities that bring more money than those small gardens.

The verbatim indicates that men prioritize participation in income-generating segments of the common bean value chain. This illuminates broader bean production and value chain activities as highly gendered. Although it reflects men’s disengagement from PVS, it also reinforced recent findings that as agricultural value chains become more commercialized, men increasingly engage and displace women from previously women-dominated roles (Pyburn et al., 2023).

Across respondent categories, 50–100% of respondents were married (Table 1). This indicates that the sample largely comprised actors living in marital unions, and marital status is reported here as contextual background rather than as an explanatory factor for varietal preferences. A sizeable proportion of women traders (10%) and consumers (23%) were widowed, which we note to signal heterogeneity among actors, although the study does not test for marital status differences in preferences given the small cell sizes. Household size also varied by respondent type: Both women and men consumers reported a larger household size (an average of 8 members) compared to their counterparts among processors and traders. This pattern is consistent with consumers having greater food provisioning responsibilities, which could in turn influence trait preferences (e.g., preference for varieties that cook faster or yield thick stew) and bean consumption behaviour (e.g., frequency of consumption).

The majority of the respondents indicated having attained primary education. However, there were more men (76%) traders with secondary education than women traders (57%). The results are consistent with several local and international reports that reveal deep-rooted gender disparities in access to education (Kebede et al., 2022; Mamadou Thierry and Ongo Emmanuel, 2023). The gender differences in education among traders align with broader empirical evidence that suggests women traders may have limited capacity to engage with formal value chain institutions and decision-making power in market negotiations. The results also showed that most of the traders—90% men and 62% women— and processors—100% women and 95% men—had off-farm employment as the primary occupation. In contrast, most consumers were primarily farmers, with all male consumers and 90% of female consumers engaged in farming. This highlights a gendered economic structure that may have implications for income-diversifying opportunities available to men and women actors in the common bean value chain.

### Varietal preferences across respondent type and sex

Table 2 presents the percentages of preferred bean varieties, disaggregated by respondent type and gender. Traders, consumers and processors exhibited relatively similar varietal preferences, with only slight variations in the ranking of preferred varieties across groups. The five most preferred bean varieties across all respondent types were NABE 4, Kanyebwa, Masindi Yellow, Nambale Omuwanvu, and NARO BEAN 1. Among these, NABE 4 emerged as the most preferred variety across all categories of respondents. Typically grown at an altitude between 1000 and 1800 meters, NABE 4 is highly marketable due to its large red mottled seeds, which produce a thick stew and superior taste (Table 3). It is also resistant to common bean diseases. This preference was echoed by information from male FGD, where one participant stated:

**Table 2:** Percentages of bean varieties preferred by men and women by respondent type.

	Consumers (%)			Traders (%)			Processors (%)		
	Total	Women	Men	Total	Women	Men	Total	Women	Men
NABE 4	40.00	38.75	42.86	44.00	40.00	48.00	33.33	100.00	30.00
NARO BEAN 1	13.00	10.13	23.81	1.33	0.00	2.00			
kanyebwa	9.57.00	10.00	2.00	5.33	0.00	11.43	9.52	0.00	10.00
Masindi yellow	10.00	8.86	9.52	33.67	38.00	24.00	9.52	0.00	10.00
NABE 14 / NAADS	5.00	5.06	4.76	2.67	2.50	2.86			
Nambale omuwanvu	5.00	3.80	9.52	4.00	5.00	2.86	19.05	0.00	20.00
NABE 6	2.00	2.00	0.00	6.67	7.50	5.71			
Small red	2.00	2.00	0.00	1.33	2.50	0			
NABE12C	1.00	1.00	0.00						
NABE17	1.00	1.27	4.76						
Black beans	1.00	1.27	0.00						
Karara	1.00	1.27	0.00	2.50	2.50	2.00			
NABE 15	1.00	1.27	0.00	1.26	0.00	2.86			
NABE 18	1.00	1.27	0.00						
NARO BEAN3	1.00	1.27	0.00						
Kadulli							4.25	0.00	4.25
NABE1							4.25	0.00	4.25
K132							4.25	0.00	4.25

**Table 3:** Consumers reasons for their preference of the selected bean varieties disaggregated by gender.

		Consumers		
Variety	Reasons for preferences (%)	Total	Women	Men
NABE 4				
	Palatable/tastes good	33.55	41.25	26.78
	Less time to cook	23.86	24.00	23.08
	Thick Stew	21.32	23.00	17.38
	Soft and not rough	11.36	12.00	7.69
	Low flatulence	3.41	2.67	8.69
NARO BEAN 1				
	Palatable/tastes good	29.55	29.63	29.41
	Less time to cook	22.73	22.22	23.53
	Thick Stew	18.18	18.52	17.65
	Soft and not rough	13.64	18.52	
	It swells	6.82	7.41	5.88
Kanyebwa				
	Palatable/tastes good	30.00	30.00	
	Less time to cook	20.00	20.00	
	Thick Stew	20.00	20.00	
	Other	15.00	15.00	
Masindi Yellow				
	Palatable/tastes good	50.00	53.85	33.33
	Less time to cook	18.75	23.08	
	Soft and not rough	12.50	7.69	33.33
	Thick Stew	12.50	7.69	33.33
	Low flatulence	6.25	7.69	

Statistical tests were not applied due to small cell sizes and low expected frequencies. The table is intended to provide indicative insights into gendered trait preferences, complementing broader patterns reported in Table 2.

We like Nambale Omumpi (NABE 4) because when it dries, it drops all the leaves. When you harvest, you only take the pods and not the leaves home, making it easy to harvest. It is also the most demanded bean on the market. Kanyebwa, because I have been eating that variety since childhood. It cooks very fast and can be kept for six months. It still cooks faster, unlike other varieties. Local schools prefer Nambale Omumpi (NABE 4) and Kanyebwa because they cook quickly and save fuel. If you do not have school fees, you can take them to school, and they will welcome you quickly. You will then use them to pay the fees.

Among consumers, NARO BEAN 1, Kanyebwa, and Masindi Yellow ranked as the second, third, and fourth most preferred bean varieties, respectively. While the majority of male consumers preferred NABE 4, NARO BEAN 1 and Masindi Yellow, a higher proportion of women (10%) than men (2%) expressed a preference for Kanyebwa.

Kanyebwa, locally referred to as the “brown-eyed beans” due to its distinctive brown eye-like marking, is widely appreciated for its good taste—comparable to that of NABE 4 and NARO BEAN 1. However, it possesses additional attributes: a shorter cooking time, as highlighted by a respondent during FGD. This trait may enhance its appeal among women, who often prioritize time-saving characteristics in meal preparation (Asiimwe et al., 2024). Kanyebwa also has a soft texture, making it suitable for a variety of local cuisines (Kiwuka et al., 2012, which may further explain its relatively higher preference among women consumers. In contrast, male consumers showed a lower preference for Kanyebwa, possibly because of its production inconsistencies. Although Kanyebwa is capable of high yields, its performance is susceptible to the appropriate use of farm inputs, resulting in significant variability, reported to range from 36 to 570 kg/ha (Mazur et al., 2011). During FGDs, male participants attributed the low yield performance of Kanyebwa to poor agronomic practices. One of the male participants noted that:

At first, I used to grow the ‘Kanyebwa’ variety. I used not to spray it, but still, the yield was not satisfactory to me due to the poor soil. I used to plant 4 kilograms, sometimes five. Out of these, the yield ranges from 50 kg to 60 kg. When the National Agriculture Advisory Services (NAADS) programme came I joined, and they started training us on the importance of spraying pests and using fertilizers to improve soil fertility. Then I started spraying the beans, and the yield has improved.

NAROBEAN1 is the second most preferred variety after NABE 4. It is a large-seeded bean released by NARO in 2010. This white to greyish variety, distinguished by its dark black stripes, is also drought-tolerant and biofortified with high concentrations of iron and zinc (Rosemary et al., 2020). These agronomic and nutritional characteristics likely contribute to its popularity among consumers.

Masindi Yellow was the second most preferred variety among traders, following NABE 4. While the majority of both male (48%) and female (40%) traders favoured NABE 4, a higher proportion of women (38%) than men (24%) preferred Masindi Yellow. Although the ranking of preferences varied, the varieties shared several desirable traits, with only slight differences across individual types. Key characteristics influencing trader and consumer preferences included good taste, thick stew and short cooking time (Table 3). Additionally, low flatulence—alongside the addition of good taste- was cited as a factor influencing consumer preference for NABE 4 and Masindi Yellow.

### Reasons for trait preferences across Value Chain Actors

#### Consumers

Table 3 presents consumers’ reasons for preferring specific common bean varieties. Palatability—particularly good taste—emerged as the primary reason for selecting NABE 4, NARO BEAN 1, Kanyebwa, and Masindi Yellow. Across all four varieties, a higher proportion of women than men consumers cited good taste and the ability to produce a thick stew as key drivers of their preferences. This finding suggests that for women, the sensory qualities of beans—especially flavour and stew consistency—play a significant role in consumers’ varietal selection (Schwartz et al., 2021; Oh et al., 2022). For instance, NABE 4, 41% of women consumers identified taste as their reason for preferring NABE 4, compared to 27% of men.

Similarly, a slightly higher proportion of women consumers (23%) than men (17%) cited thick stew as a reason for preferring NABE 4. In contrast, more men (9%) than women (3%) mentioned low flatulence as a key consideration in their choice. NARO BEAN 1 was preferred by nearly equal proportions of male and female consumers, primarily due to its good taste. Additionally, many consumers favoured NARO BEAN 1 because of its short cooking time, which likely makes it more economical for households with limited financial resources (Nafula et al., 2022). Consumers also noted that NARO BEAN 1 has a soft texture, making it suitable for diverse culinary preparations. Moreover, consumers explained that NARO BEAN 1 “swells” during cooking, enabling it to feed large families without increasing food cost.

For Kanyebwa, only women responded to the question about their preferences for the trait. A majority indicated that they preferred the variety due to its good taste, shorter cooking time, and the ability to produce a thick stew. These preferences reflect women’s dominant role in household food preparation, where cooking efficiency and sensory appeal directly influence their trait preferences to reduce labour burden and family satisfaction. Similarly, more women (53%) than men (50%) preferred Masindi Yellow for its taste. However, a higher proportion of women cited thick stew as their main reason for preference, while slightly more men (8%) than women (6%) valued the variety because of its low flatulence. NABE 4 was preferred for its taste a significantly larger proportion of women (75%) consumers compared to men (25%).

Overall, taste, stew thickness, and cooking time were the primary traits that influenced consumers’ choice of common bean varieties. The results indicate that the five most preferred varieties shared key traits—namely, good taste, shorter cooking time, and stew thickness. NABE 4, NARO BEAN 1, and Masindi Yellow were also described as “soft and not rough”, a texture-related characteristic that the consumers found appealing. Gender-disaggregated responses demonstrated that more women than men considered the stew thickness as an important criterion in their varietal selection. For instance, 20% of the women compared to 15% of the men preferred NABE 4 due to its thick stew. Similarly, 19% of women and 18% men mentioned thick stew as a reason for preferring NARO BEAN 1. Both NABE 4 and Masindi Yellow were additionally valued for their low flatulence, a trait that further enhanced their appeal among consumers.

The findings suggest that sensory traits of common bean grain—such as taste and stew thickness—extend beyond personal preferences as they may reflect socio-cultural gender roles around food preparation and household nutrition. Breeding programmes that ignore such traits may develop varieties that face low adoption across the value chain, even if agronomic performance is high. Importantly, trait preferences related to labour-saving (e.g., short cooking time) and nutrition are particularly salient for women who are primarily more involved in food preparation at the household level and are predominantly involved in common bean production. Therefore, the convergence between nutrition and time-use burdens should be considered by breeders and seed distributors.

#### Traders

Table 4 presents traders’ preferences for the various common bean varieties. Customer demand emerged as the primary reason for traders’ preferences for NABE 4, Masindi Yellow, NABE6, and Kanyebwa. While consumers (as shown in Table 3) prioritized traits such as taste, thick stew, and short cooking time, traders emphasized factors related to marketability—specifically, affordability and local availability of the preferred varieties. These varieties were commonly sourced from farmers and easily resold in local Ugandan markets. As intermediaries in the value chain, traders prioritize traits that reduce transaction costs (e.g., availability and affordability) and enhance customer demand. For example, NABE 4 and Masindi Yellow are popular among customers and have relatively stable supply chains, which guarantee turnover and minimize unsold inventory. On the other hand, NABE 4 and NABE 6 have gained popularity in local markets due to their introduction by NARO, largely because of their drought tolerance and resistance to common bean diseases (Gerald et al., 2015). Kanyebwa, also widely available and affordable, is likely preferred by traders due to its strong appeal among women consumers (Table 2), further enhancing its market demand. The traders also noted a preference for NABE 4 and Masindi Yellow due to their strong performance in the national market. At the same time, NABE 6 and Kanyebwa were favoured for their longer shelf life and resistance to bean weevils, respectively.

**Table 4:** Traders’ reasons for their preference for the selected bean varieties disaggregated by gender.

		Traders (*N* = 75)		
Variety	Reasons for preferences (%)	Total	Women	Men
NABE 4				
	Highly preferred by customers	31.88	29.41	85.71
	Affordable	11.59	14.71	8.57
	Available	20.29	14.71	25.71
	National market	32.14	17.65	8.57
Masindi Yellow				
	Highly preferred by customers	36.54	36.11	37.5
	Affordable	11.54	13.89	6.25
	Available	17.31	16.67	18.75
	National market	13.46	16.67	
NABE 6				
	Affordable	30.77	33.33	25
	Available	30.77	22.22	50
	highly preferred by customers	23.08	22.22	25
	Longer shelf life	7.69	11.11	
Kanyebwa				
	Highly preferred by customers	33.33		33.33
	Affordable	22.22		22.22
	Available	22.22		22.22
	Resistance to bean weevil	11.11		11.11

The findings on traders’ preferences indicate that their varietal preferences are mediated by both intrinsic bean traits and their market knowledge and customers’ feedback. Strong preference for Masindi Yellow by women traders may be attributed to the predominant role in local markets and understanding of what women consumers prefer when selecting common bean grain for consumption. The convergence of traits preferred by consumers and traders emphasizes the need for common bean breeders to prioritize traits that respond to both market demand and gender-specific preferences for bean products. This is critical in enhancing both adoption and profitability across the value chain and the eventual consumption of bean grain.

#### Processors

Table 5 presents reasons processors preferred NABE 4, Nambale Omuwanvu, Kanyebwa, and Masindi Yellow for processing bean products such as flour, snacks, and pre-cooked whole grains. Key attributes influencing their choices included palatability, price, colour, and grain size. Except for one female processor, all other processors were men, and the majority expressed preference for NABE 4, Kanyebwa, Nambale Omuwanvu, and Masindi Yellow primarily due to their good taste and favourable price. These preferences were closely aligned with consumers’ motivations for selecting the same varieties, suggesting that processors may be influenced by consumer demand. Although other attributes—such as grain size and colour—were also mentioned, their influence appeared less significant compared to taste. This alignment implies that processors possess a relatively strong understanding of factors shaping consumers’ preferences for common bean products and their role in producing marketable and palatable products for consumers. Additionally, attributes favoured by processors—taste, soft texture, and short cooking time—reduce processing costs (e.g., energy or fuel use) and align products with consumer expectations, thereby improving profitability.

**Table 5:** Processors’ reasons for their preference for the selected bean varieties disaggregated by gender.

		Processor		
Variety	Reasons for preferences (%)	Total (*n* = 21)	Women (*n* = 1)	Men (*n* = 20)
NABE 4				
	Taste	46.67	50.00	50
	Price	13.33	50.00	50
Nambale Omuwanvu				
	Taste	50		50
	Other	25		25
	Resistance to bean weevil	12.5		12.5
	Colour	12.5		12.5
Kanyebwa				
	Taste	50		50
	Price	25		25
	Colour	25		25
Masindi Yellow				
	Taste	66.67		66.67
	Size	33.33		33.33

Processors are closely linked to consumers, who often provide feedback on desirable bean product characteristics. Consequently, it is unsurprising that processors’ preferences align closely with those of consumers, as shown in Table 3. Gerald et al. (2015) reported that both NABE 4 and Nambale Omuwanvu are early maturing and highly marketable varieties widely cultivated across Uganda because of consumer demand. However, while prior studies have focused on agro-ecological and production-related traits, the present finding aligns with those of Buzera et al. (2018), who also observed strong convergence between consumer and processor preferences, particularly for traits such as taste, cooking time, soft grain coat, and texture.

The findings indicate alignment between processors and consumers’ varietal and trait preferences, which reflects transmission of demand-driven attributes of common bean products along the value chain. This underscores that processors respond to consumer preferences by anticipating market demands through the selection of common bean varieties that possess favourable sensory and processing qualities. The observed varietal preferences for NABE 4, Kanyebwa, and Masindi Yellow and trait congruence across all studied market actors underscore the importance of demand-driven and market-led breeding efforts. These findings imply that current development breeding efforts are likely to be co-informed by processors and traders, given their downstream role of bridging production and consumption of common bean products.

Notable convergence and divergence of farmer trait preferences and those expressed by downstream actors are observed. Reduced cooking time. Common traits that traders, processors, and consumers prioritized are valued by farmers, especially women (Nchanji et al., 2021; Asiimwe et al., 2024). Traits that are increasingly prioritized by farmers as common beans become more commercialized—marketability, taste, grain size, and prices (Nchanji et al., 2023)—were also highlighted by traders, processors and consumers. While actors further along the value chain emphasized post-harvest qualities, including taste, stew thickness, these traits are less prioritized by farmers because of the differences in constraints at production levels and objectives of bean farming. Farmers’ primarily focus on traits that ensure a large harvest and a quicker turnaround time to attend to household expenses and pay for inputs may outweigh the benefits of prioritizing post-harvest qualities of bean grain valued by traders and processors. Conversely, yield and climate resilience traits may be indirectly important for traders and processors as they affect the supply and quality of grain (Asfaw et al., 2012). These observations point to the need for breeders to balance agronomic traits and end-user traits to ensure that varieties succeed in the farm, market, and consumer households.

### Socioeconomic variables and varietal preferences

Table 6 presents descriptive patterns between socioeconomic characteristics and gender varietal preferences among traders. Given the small cell sizes, these patterns are interpreted cautiously and are presented primarily to generate hypotheses for future studies. Educational attainment was common among both men’s and women’s preferences for NABE 4, with 81% of male and 82% of female traders who preferred the variety having attained secondary or higher. Because traders in the sample were generally relatively educated, this distribution may partly reflect sample composition; nonetheless, it is consistent with the idea that education could shape traders’ strategic decisions, such as opting for widely available varieties like NABE 4 to minimize transaction costs associated with sourcing less common bean types. Additionally, traders with higher education levels may have a better understanding of consumer preferences, which could inform their choice to trade in NABE 4.

**Table 6:** Association between socio-economic factors and traders’ bean variety preferences by gender.

Variety	Variable	Response	Women	Men
NABE 4	Education (%)	Primary	18.75	17.65
		Secondary or higher	81.25	82.35
	Marital status (%)	Never married	25	47.06
		Married	62.5	47.06
		Widowed	6.25	
		Cohabiting	6.25	
		Separated		5.88
	Household headship (%)	Spouse	68.75	29.41
		Head	31.25	70.59
	Occupation (%)	Farming	6.67	13.33
		Off-farm	93.33	86.67
Masindi Yellow	Education (%)	Primary	31.25	42.86
		Secondary or higher	68.75	57.14
	Marital status (%)	Never married	25	14.29
		Married	62.5	71.43
		Widowed	12.5	
		Cohabiting	0.00	
		Separated		14.29
	Household headship (%)	Spouse	68.75	14.29
		Head	31.25	85.71
	Occupation (%)	Farming		28.57
		Off-farm	100	71.43

A similar distribution was observed for Masindi Yellow, with 68% male traders and 57% of female traders who preferred the variety reporting secondary or higher education, although this may largely reflect the overall education profile of traders in the sample rather than a robust education-preference association. Even so, Masindi Yellow remains a local variety rather than a newly promoted improved type, and its continued popularity may be attributed to its consistent availability in the market, as farmers have sustained its cultivation despite the widespread introduction and promotion of newer varieties. Masindi Yellow’s appeal may lie in its desirable traits, particularly its good taste and ease of cooking, which enhance its attractiveness to consumers.

In this sample, most traders were engaged in off-farm occupations, and the higher share of NABE 4 and Masindi Yellow preferences observed among married and off-farm traders should therefore be read as descriptive patterns that may partly reflect the underlying composition of the trader group rather than a distinct marital-or occupation-specific effect. While off-farm engagement and marital status could plausibly shape market exposure and information access, the small cell sizes and descriptive design do not allow us to attribute these differences to occupation or marital status. We therefore present these distributions mainly to contextualize who is represented among traders and to flag potential heterogeneity that could be explored in larger samples, rather than to make strong claims about within-household autonomy or occupational mechanisms.

Table 7 presents the descriptive distributions between socioeconomic variables and processors’ preferences of bean varieties disaggregated by gender. Due to the participation of only one woman processor, the interpretation of women’s results is excluded. Among male processors, those with lower educational attainment more frequently reported preferring NABE 4 compared to their more educated counterparts. This preference pattern relates to the widespread cultivation and market availability of NABE 4, coupled with lower awareness among less-educated processors of alternative varieties with superior processing traits. Marital status and engaging in off-farm activities were more commonly observed among male processors’ preferences for NABE 4 and NARO Bean 1, possibly due to increased exposure to supply channels and market information. Additionally, both male household heads and spouses showed similar likelihoods of preferring NARO Bean 1, whereas being a household head was more commonly observed among those who preferred NABE 4.

**Table 7:** Association between socio-economic factors and processor bean variety preferences by gender.

Variety	Variable	Response	Women	Men
NABE 4	Education (%)	Primary	100	71.43
		Secondary or higher		28.57
	Marital status (%)	Never married		42.86
		Married	100	57.14
		Cohabiting		0.00
	Household headship (%)	Spouse	100	
		Head		100.00
	Occupation (%)	Farming		
		Off-farm	100	100.00
NARO Bean 1	Education (%)	Primary		81.25
		Secondary or higher		18.75
	Marital status (%)	Never married		25.00
		Married		75.00
		Cohabiting		0.00
	Household headship (%)	Spouse		50.00
		Head		50.00
	Occupation (%)	Farming		0.00
		Off-farm		100.00

Table 8 presents the descriptive distribution between gender-disaggregated consumers’ preferences and socioeconomic characteristics for the two most preferred bean varieties—NABE 4 and NARO Bean 1. Higher educational attainment was positively associated with preferences for both varieties. Women with primary education demonstrated greater preferences for NABE 4 and NARO Bean 1 compared to those with no formal education. A similar trend was observed among men: those with primary, secondary, or higher education showed a stronger preference for NABE 4 and NARO Bean 1 compared to those without any educational qualification. While the widespread cultivation and availability of NABE 4 may partly explain its popularity, it is likely that more educated consumers, both men and women, were more aware of the nutritional benefits of beans, including their role as an affordable source of protein. NARO Bean 1, which was promoted for its high nutritional content following its release in 2016 (Alliance of Bioversity International and CIAT, 2016), may have attracted educated consumption consumers who were more exposed to health-related information.

**Table 8:** Association between socio-economic factors and consumer bean variety preferences by gender.

Variety	Variable	Response	Women (34)	Men (9)
NABE 4	Education (%)	No formal education	5.88	
		Primary	73.53	55.56
		Secondary or higher	20.59	44.44
	Marital status (%)	Married	58.82	100
		Divorced	0.00	
		Separated	5.88	
		Widowed	32.35	
		Cohabiting	2.94	
	Household headship (%)	Spouse	8.82	
		Head	91.18	100.00
	Occupation (%)	Farming	91.18	100.00
		Off-farm	8.82	
NARO Bean 1	Education (%)	No formal education	0.00	
		Primary	50.00	80.00
		Secondary or higher	50.00	20.00
	Marital status (%)	Married	87.5	80.00
		Divorced	12.5	
		Separated	0.00	
		Widowed	0.00	
		Cohabiting	0.00	20.00
	Household headship (%)	Spouse	12.5	
		Head	87.50	100.00
	Occupation (%)	Farming	87.5	100.00
		Off-farm	12.5	

Preference for NABE 4 was higher among married consumers of both genders, with 59% of married men and 100% of women indicating a preference for the variety. Moreover, 32% of widowed women consumers reported a preference for NABE 4. Similarly, preferences for NARO Bean 1 were more pronounced among married consumers, with 80% of married men and 85% of married women expressing a preference for the variety. These patterns may reflect the influence of household size and food provisioning responsibilities, as married individuals are more likely to belong to larger households and may prioritize readily available and affordable bean grain. In addition, the strong preferences of NARO Bean 1 among married consumers may also be influenced by concerns about household nutrition, given the variety’s promoted attributes relate to iron and zinc content.

Women’s and men’s preferences for NABE 4 and NARO Bean 1 also appeared to align with household headship. Household heads of both sexes are more likely to exercise autonomy in food consumption decisions, which may influence their preference for locally available and affordable bean varieties that meet household consumption needs. Primary occupation was similarly associated with varietal preferences: all male consumers and 91% of women consumers who preferred NABE 4 identified farming as their primary occupation. This suggests crop production decisions are closely tied to self-sufficiency goals in bean consumption. The findings further indicate dietary differences between farmers and non-farmers, with farmers preferring to consume bean varieties they produce while non-farmers tend to choose alternative varieties.

In summary, preferences NABE 4, NARO BEAN 1, Masindi Yellow, Kanyebwa and Nambale Omuwanvu were influenced by trait characteristics, gendered roles, market access, and knowledge asymmetries. While traders of common bean grain favoured bean varieties with traits tied to customer demand and inventory turnover, processors preferred varieties with traits aligned with sensory attributes that appeal to consumers and ease of processing. Consumers emphasized taste, stew thickness, and shorter cooking time, particularly among women. These variations highlight the importance of breeding programmes to conduct participatory value chain analysis in order to recognize actor and gendered varietal and trait preferences and integrate them in the development of inclusive and commercially viable product profiles.

## Conclusion and recommendation

This study provides critical insights into varietal and trait preferences among bean value chain actors in Uganda—specifically, traders, consumers, and processors—by applying a gender lens. From this analysis, it is important to note that what consumers prefer drives preference along the value chain. NABE 4 was also popular among processors, who have consistently shown a preference for the varieties that consumers like. For processors, cooking qualities such as thick stew mattered to most processors. This was also similar among consumers who preferred fast cooking and thick stew. However, most traders stocked these beans because customers preferred them and because there was a national demand for such bean varieties.

Top five bean varieties preferred across the three respondent types were NABE 4, NARO BEAN 1, Masindi Yellow, KANYEBWA and Nambale Omuwanvu. These varieties were preferred because of their good taste, thick stew, less cooking time, colour and low flatulence. Therefore, common bean breeders should target these varieties and the various traits to boost household food security. Moreover, the variety can also help empower women, given that more women participate in the common bean value chain as traders.

The preferences expressed by consumers, traders, and processors in this study reveal both convergences and divergences from those typically emphasized by farmers in participatory varietal selection exercises. For instance, traits such as high yield and disease tolerance—often prioritized by farmers—were less frequently cited by consumers or traders. Instead, actors further along the value chain emphasized post-harvest qualities, including taste, stew thickness, cooking time, and market demand. This divergence suggests the need for breeders to balance agronomic performance with end-user traits to ensure that varieties are not only adopted at the farm level but also succeed in markets and households.

However, the study reveals that while all actors universally preferred good taste, thick stew, and short cooking time, the reasons behind these preferences and the varietal choices showed important gendered distinctions. Women consumers, for instance, demonstrated a stronger preference for varieties like Kanyebwa due to its fast-cooking time and thick stew traits aligned with their domestic roles. Male traders and processors were more likely to prioritize market demand, shelf life, and size. The information should guide breeders to understand not only men’s and women’s traits and varietal preferences but also value chain-wide preferences.

Gender also appeared to shape varietal preferences through socio-demographic factors. Given the exploratory design and small cell sizes in some subgroups, these patterns should be interpreted cautiously and treated as hypothesis-generating. Women with primary education and household decision-making authority were more frequently reported as preferring improved varieties like NABE 4 and NARO BEAN 1. At the same time, men’s preferences were associated with market positioning and their roles as household heads. Similarly, women traders’ trait preferences and choices of varieties to trade were observed to vary across categories of being household heads and educational attainment. These findings reinforce the importance of understanding how social roles, household responsibilities, and education may shape varietal choices among women and men across the value chain. These have implications for breeders and policymakers may need to go beyond aggregate preferences and attend to gender-differentiated needs by integrating gendered insights into breeding pipelines and market development strategies.

While breeders have previously emphasized productivity, drought tolerance, and disease-resistant traits, these socio-demographic factors also need consideration. There is a need to ensure that improved bean varieties are not only technically superior but also socially responsive, thereby supporting an inclusive common bean value chain. Furthermore, not only did the study show both alignment and misalignment of traders, processors, and consumers’ varietal and trait preferences, but findings also showed both convergence and divergence between farmer trait preferences and those of downstream actors. While some traits (e.g., reduced cooking time and marketability) were commonly valued by common bean actors, post-harvest attributes emphasized by traders, processors, and consumers are often deprioritized by farmers. Therefore, breeding programmes in Uganda and sub-Saharan Africa should adopt a participatory, market-oriented approach to ensure that product profiles balance supply- and demand-side traits.

## Data Availability

The raw and processed datasets, survey instruments, and the data dictionary containing variable definitions, coding schemes, and details of constructed variables are available in the Harvard Dataverse repository (https://doi.org/10.7910/dvn/earxxh) and can also be accessed via https://hdl.handle.net/10568/168218. The repository contains the full underlying data and metadata needed to reproduce the tables and verify the findings.
